# Psychological well-being and academic performance of Ukrainian medical students under the burden of war: a cross-sectional study

**DOI:** 10.3389/fpubh.2024.1457026

**Published:** 2025-01-06

**Authors:** Mykhaylo Korda, Arkadii Shulhai, Oksana Shevchuk, Oleksandra Shulhai, Anna-Mariia Shulhai

**Affiliations:** ^1^Department of Medical Biochemistry, I.Ya. Horbachevsky Ternopil National Medical University, Ternopil, Ukraine; ^2^Department of Public Health and Healthcare Management, I.Ya. Horbachevsky Ternopil National Medical University, Ternopil, Ukraine; ^3^Department of Pharmacology and Clinical Pharmacology, I.Ya. Horbachevsky Ternopil National Medical University, Ternopil, Ukraine; ^4^Department of Children’s Diseases and Pediatric Surgery, I.Ya. Horbachevsky Ternopil National Medical University, Ternopil, Ukraine; ^5^Department of Pediatrics №2, I.Ya. Horbachevsky Ternopil National Medical University, Ternopil, Ukraine; ^6^Department of Medicine and Surgery, University of Parma, Parma, Italy

**Keywords:** mental health, medical students, stress, anxiety, depression, academic performance, Ukraine

## Abstract

**Introduction:**

The mental health of medical students is a key factor for academic performance and the delivery of high-quality medical care in the future. Globally, medical students face numerous challenges that can affect their education. Living and studying facing the war has a crucial influence on medical students’ education and daily life. The study aimed to determine the psychological well-being and academic achievements of Ukrainian medical students who lived in the rear areas after the 18 months of the Russian–Ukrainian war.

**Methods:**

The cross-sectional study was conducted at I.Ya. Horbachevsky Ternopil National Medical University. We analyzed the psychological well-being and educational achievements of medical students from the second to fifth year of Medical Faculty using the self-report scales DASS-21 and IES-R. A total of 776 students filled out the DASS-21 scale and 491 IES-R scale, respectively.

**Results:**

Following the first 18 months of the war, 62.5, 59.6, and 58.8% of Ukrainian medical students self-reported signs of stress, anxiety, and depression, respectively. Severe and extremely severe depressive symptoms were observed in 25.6% of students, anxiety in 31.9%, and stress in 29.7%, with a higher prevalence among female students. Additionally, 44.2% of responders had significant symptoms of post-traumatic stress disorder, with the highest scores on the hyperarousal subscale (>37). Academic performance in 2023 was significantly lower compared to the pre-war period in 2019 (*p* < 0.001) (*p* = 0.000). The multiple linear regression analysis showed that academic performance was positively associated with depression (*p* = 0.003), hyperarousal (*p* < 0.001), anxiety (*p* = 0.03), and negatively associated with stress (*p* = 0.002).

**Conclusion:**

Ukrainian medical students in rear areas are facing various challenges, with war-related stressors having a profound impact on their mental health. Our findings have shown an increasing prevalence of anxiety, stress, depression, and symptoms of post-traumatic stress disorder, all of which may negatively affect academic performance. The educational process during wartime is important for facilitating qualified medical personnel and quality healthcare. Understanding the burden of war allows for the development of wellness programs that support student mental health in conflict zones.

## 1 Introduction

Public health has been profoundly impacted by the full-scale Russian invasion of Ukraine, affecting both the frontline and rear regions of the country. Constant threats to life and health, including military operations, missile strikes, and the psychological stress resulting from these experiences, contribute to trauma, anxiety, and depression. War and military conflicts are crucial predictors of anxiety and depressive disorders due to a high incidence of trauma to both mental and physical health (1). The impact of war on psychological well-being is complex. It can be determined by the number, structure, and duration of factors such as direct trauma and/or psychosocial stressors and their levels, socio-economic status, and social functioning (2). Moreover, emotional suffering associated with war can arise from direct exposure and indirect sources, such as witnessing scenes of war on television or social media (2–4). According to the indirect exposure theory, individuals who are concerned about war but live outside the conflict zone may suffer negative mental health consequences, similar to those on the frontline (4).

War has a profound effect on mental health, with the development and exacerbation of mental disorders becoming increasingly widespread across the country. Among them the most common are anxiety and generalized anxiety disorder, post-traumatic stress disorder (PTSD), and depression (1, 5). Previous studies have identified manifestations of mental disorders among people of different age groups from both frontline and rear regions in Ukraine (1, 2, 4, 6, 7). For instance, the anxiety prevalence ranged from 23.7 to 94.9%, PTSD from 7.6 to 68.9%, and depression from 4.1 to 41.2% in Ukrainian youth (2). Moreover, some studies have reported a higher prevalence of PTSD in women compared to men, suggesting gender disparity (4, 6), which contrasts with other studies (8), highlighting the need for further research.

One of the important factors that determine population health status, estimated at 10–15%, is medical factors that have been associated with the provision of medical care. High-quality medical care requires a high qualification of healthcare professionals and a sufficient level of theoretical and practical training. The quality of education for future healthcare workers is a direct reflection of their qualifications. The physical and psychological well-being of students have been recognized as an important component in achieving educational success (3, 9). American studies indicate that the rigorous nature of medical school training presents numerous challenges, which negatively impact mental health and sleep habits, even more so than the effects of the COVID-19 pandemic (10). However, medical students already face many stressors that can affect their psychological well-being, and war contributes as an additional factor affecting their mental health.

Poor psychological well-being of young people can have a negative impact on academic performance and competency acquisition. It can lead to a decrease in empathy, humanity, compassion, patience, and other essential qualities of medical students that are crucial for future successful medical practice (5, 11). Additionally, untreated mental health issues among medical students are thought to be a major contributing factor to poor mental health among practicing physicians, which in turn affects the quality of the healthcare system (12).

Improvement and adaptation of the organization of medical students’ education and training during the war are highly required (13). It is important for educational institutions to understand the mental health burden of the war on medical students (14) and how it impacts their academic achievements and further training.

The study aimed to assess the psychological well-being and academic performance of Ukrainian medical students living in rear areas after 18 months of Russia’s war against Ukraine.

## 2 Materials and methods

### 2.1 Data collection

This cross-sectional study was conducted at I.Ya. Horbachevsky Ternopil National Medical University, Ternopil, Ukraine. The study examined the psychological well-being and academic performance of medical students from the second to fifth year of the medical faculty. The “Depression, Anxiety and Stress” scale (DASS)-21 (15, 16) was used to assess signs of depression, anxiety, and stress, while the Impact of Event Scale-Revised (IES-R) was used to define symptoms of post-traumatic stress (17, 18). The questionnaires also contained questions about demographic data, such as age, gender, year of study, and place of residence. A virtual approach was used to encourage student participation. All students from the second to fifth year of medical faculty received an invitation email with a link to anonymous online surveys. The email emphasized that participation was voluntary. Students were informed that the questionnaire was anonymous and confidential and that there were no correct or wrong answers to the questions. A total of 776 students, 180 males and 596 females, completed the self-report scale DASS-21, while 491 students completed the self-reported IES-R scale. All participants were over 18 years of age and provided online informed consent for their involvement in the survey. Obtaining informed consent from all participants and completing the questionnaires was the main condition for inclusion in the study. Students who did not provide consent were excluded from the study.

To compare the academic achievements of medical students in 2019 and 2023, we analyzed the grade point averages (GPAs) from the autumn semesters of 2019 and 2023. This analysis categorized the GPAs according to the students’ year in medical school, using data provided by the dean’s office of the medical faculty. For this comparison, we considered the absence of changes in personnel, curriculum, and main teaching methods.

The Ethical Committee of the I.Ya. Horbachevsky Ternopil National Medical University reviewed and approved the study (approval number №9/2023).

### 2.2 Survey questionnaires

The DASS-21 scale consisted of 21 items, which covered three self-assessment scales designed to measure emotional states of depression, anxiety, and stress. Each scale contained seven items, divided into subscales based on similar content. The DASS-21 rating scale in digital value included the following: 0 – did not apply at all, 1 – applied to a certain extent or for a certain time, 2 – applied to a large extent or for a large part of the time, and 3 – applied very often. The scale was characterized by the high-reliability coefficient (Cronbach’s alpha): [depression (*α* = 0.92), anxiety (*α* = 0.95), and stress (*α* = 0.87)]. Based on the results of the questionnaire, the levels of depression, anxiety, and stress were classified as normal, mild, moderate, severe, and extremely severe (15, 16). According to the manual for depression scale: 0–4 (normal), 5–6 (mild), 7–10 (moderate), 11–13 (severe), ≥14 (extremely severe); anxiety: 0–3 (normal), 4–5 (mild), 6–7 (moderate), 8–9 (severe), ≥10 (extremely severe); and stress: 0–7 (normal), 8–9 (mild), 10–12 (moderate), 13–16 (severe), ≥17 (extremely severe).

The IES-R was developed to measure subjective distress caused by traumatic events. In this scale, the symptoms of PTSD are characterized by general subjective stress and three subscales: intrusion – 8 points, avoidance – 8 points, and hyperarousal – 6 points. The questionnaires were evaluated according to the Likert scale, where 0 – no symptoms, 1 – some symptoms, 2 – the moderate presence of symptoms, 3 – quite a bit presence of symptoms, and 4 – the extreme presence of symptoms. The reliability coefficient for IES-R (Cronbach’s alpha) has defined *α* = 0.91. Based on the total score for all 22 IES-R items, the presence of PTSD was interpreted as follows: up to 24 points – no symptoms of PTSD, 24–32 points – partial symptoms of PTSD, 33–37 points – probable diagnosis of PTSD, more than 37 points – significant PTSD symptoms (17, 18).

### 2.3 Statistical analysis

Statistical analysis of the obtained results was conducted using the SPSS 21.0 software. Descriptive statistics, such as mean (M) and standard deviation (SD), were used to determine the prevalence and severity of stress, anxiety, depression, post-traumatic stress disorder, and students’ academic performance. Frequency distributions from a set of descriptive statistical methods were used in this study: an absolute value (*n*) and percentage amount (%). The frequency scores between groups were compared using Pearson’s chi-square (χ2) test. The quantitative parameters of the observation groups were compared using the student’s *t*-test. Pearson’s correlation analysis was performed to assess the correlation between academic achievements and stress, anxiety, depression, and the presence and severity of PTSD.

Multiple regression analysis was carried out to determine the factors that influenced medical student academic achievements in Ukrainian rear regions during the war period. This involved evaluating independent predictors that affect students’ education. For this, a multiple linear regression model was formed, with GPA as the dependent variable and depression, anxiety, stress, avoidance, intrusion, and hyperarousal as independent variables. This allowed us to find the theoretical value of the level of academic attainment according to regression coefficients and the values of risk factors on a nominal, ordinal, or quantitative scale that affect academic success.

Differences between values were considered significant at a *p*-value of <0.05.

## 3 Results

The prevalence of depression, anxiety, and stress among medical students of Western Ukraine after 18 months of war, according to analysis of the DASS-21 scale, is shown in Table 1. Among responders, 62.5% showed signs of stress, 59.6% of anxiety, and 58.8% of depression. Severe and extremely severe depressive symptoms were found in 25.6%, anxiety in 31.9%, and stress in 29.7% of students. The comparison of depression, anxiety, and stress incidence among male and female medical students revealed that these symptoms were more common in women than men. Comparing gender prevalence of anxiety and stress symptoms, it was found that the level of anxiety was 1.54 times higher (*p* = 0.001), and the level of stress was 1.43 times higher (*p* = 0.001) among the female students due to severe and extremely severe manifestations (Table 1).

**Table 1:** Prevalence of depression, anxiety and stress levels among the medical students of Western Ukraine after 18 months of war based on DASS-21 scale.

Level of severity	Total population, *n* = 776		Males, *n* = 180		Females, *n* = 596		*р*-value	
	*n*	%	*n*	%	*n*	%		
Depression								
Normal	320	41.2	82	45.6	238	39.9	0.179	0.001*
Mild	117	15.1	27	15.0	90	15.1	0.973	
Moderate	140	18.1	27	15.0	113	19.0	0.226	
Severe	106	13.7	21	11.6	85	14.3	0.374	
Extremely severe	93	11.9	23	12.8	70	11.7	0.708	
Anxiety								
Normal	314	40.5	100	55.6	214	35.9	0.001*	0.001*
Mild	122	15.7	25	13.8	97	16.3	0.441	
Moderate	92	11.8	13	7.2	79	13.2	0.028*	
Severe	58	7.5	16	8.9	42	7.1	0.410	
Extremely severe	190	24.5	26	14.5	164	27.5	0.001*	
Stress								
Normal	291	37.5	88	48.9	203	34.1	0.001*	0.001*
Mild	111	14.3	20	11.1	91	15.3	0.162	
Moderate	143	18.4	29	16.1	114	19.1	0.360	
Severe	185	23.8	34	18.9	151	25.3	0.075	
Extremely severe	46	5.9	9	5.0	37	6.2	0.547	

* *p* < 0.05.

According to the DASS-21 scale, the level of stress in medical students was 9.35 ± 4.63, anxiety 5.78 ± 4.74, and depression 6.74 ± 4.90 (Table 2). Quantitative characteristics of stress levels exceeded the anxiety level by 1.61 times (*p* < 0.001) and the depression level by 1.39 times (*p* < 0.012). Comparison of these quantitative characteristics between the men and women showed significant gender differences in anxiety (*p* = 0.000) and stress (*p* = 0.001) levels (Table 2).

**Table 2:** Average scores of depression, anxiety, and stress among the medical students of Western Ukraine after 18 months of war according to the DASS-21 scale (M ± SD).

Subscale	Surveillance group	Value	*р*-value
Depression	Total population	6.74 ± 4.90	
	Males	6.33 ± 5.01	0.197
	Females	6.87 ± 4.86	
Anxiety	Total population	5.78 ± 4.74	
	Males	4.28 ± 4.39	0.000*
	Females	6.21 ± 4.75	
Stress	Total population	9.35 ± 4.63	
	Males	8.34 ± 4.76	0.001*
	Females	9.66 ± 4.56	

Based on DASS-21, depression scale: 0–4 (normal), 5–6 (mild), 7–10 (moderate), 11–13 (severe), ≥14 (extremely severe); anxiety: 0–3 (normal), 4–5 (mild), 6–7 (moderate), 8–9 (severe), ≥10 (extremely severe); stress: 0–7 (normal), 8–9 (mild), 10–12 (moderate), 13–16 (severe), ≥17 (extremely severe). * *p* < 0.05.

Analysis of the post-traumatic stress disorder prevalence according to the results of the IES-R questionnaire (Table 3) showed that 44.2% (*р* = 0.001) of all medical students had high scores (>37 points) and significant symptoms of PTSD. The prevalence of PTSD manifestations was 45.61% among male students and 72.41% among female students, indicating that female students were 1.58 times more likely to experience PTSD symptoms compared to male students (*p* = 0.001). Moreover, significant PTSD symptoms were 2.54 times more frequent in women than in men (*p* < 0.001).

**Table 3:** Prevalence of post-traumatic stress disorder among the medical students of Western Ukraine after 18 months of war according to the IES-R scale (*n* = 491).

Values	Surveillance group	*n*	% (95% CI)	*р*-value
Up to 24 points (no symptoms of PTSD)	Total population	167	34.1 (31.5–36.7)	
	Males	62	54.3 (50.1–58.4)	0.000*
	Females	105	27.6 (24.9–31.2)	
24–32 points (partial symptoms of PTS)	Total population	72	14.7 (13.8–15.5)	
	Males	21	18.4 (16.2–20.7)	0.195
	Females	51	13.5 (12.8–14.3)	
33–37 points (probable diagnosis of PTSD)	Total population	35	7.1 (6.7–7.5)	
	Males	8	7.0 (6.4–7.6)	0.582
	Females	27	7.2 (6.6–7.6)	
More than 37 points (significant PTSD symptoms)	Total population	217	44.2 (41.8–46.6)	
	Males	23	20.2 (18.9–21.5)	0.000*
	Females	194	51.4 (46.7–56.2)	

CI, confidence interval; * *p* < 0.05.

The IES-R questionnaire also allows for the analysis of three PTSD subscales after 18 months of war: intrusion, avoidance, and hyperarousal. The highest score (1.61 ± 0.37) was determined in the hyperarousal subscale (Table 4). Female students showed significantly higher scores than male students across all IES-R subscales.

**Table 4:** Quantitative characteristics of the post-traumatic stress disorder components among the medical students of Western Ukraine after 18 months of war according to the IES-R scale (M ± SD).

Subscale	Surveillance group	Value index	*р*-value
Intrusion	The whole group	1.48 ± 0.26	
	Men	1.11 ± 0.27	0.004*
	Women	1.59 ± 0.27	
Avoidance	The whole group	1.45 ± 0.28	
	Men	1.10 ± 0.29	0.009*
	Women	1.55 ± 0.29	
Hyperarousal	The whole group	1.61 ± 0.37	
	Men	1.23 ± 0.43	0.043*
	Women	1.72 ± 0.36	

* *p* < 0.05.

A comparison of academic performance among second to fifth-year medical students who studied in 2019 (before the war) and medical students in 2023 (after 18 months of war) (Table 5) was performed to establish the relationship between the medical students’ academic achievement and mental health. It was defined that medical students’ academic performance in 2023 was significantly lower compared to 2019, regardless of the year of study (*p* < 0.001).

**Table 5:** Comparative characteristics of medical student academic performance in 2019 and 2023 (M ± SD).

Year of study	Academic year	Number of students	Grade point average (GPA)	*р*-value
2nd year	2023	321	162.05 ± 8.67	0.000*
	2019	198	166.93 ± 5.26	
3rd year	2023	259	157.13 ± 9.50	0.000*
	2019	398	164.17 ± 6.48	
4th year	2023	380	154.07 ± 8.84	0.000*
	2019	347	164.75 ± 5.89	
5th year	2023	344	159.75 ± 7.49	0.000*
	2019	376	164.35 ± 5.33	

* *p* < 0.05.

Significant inverse correlations between the levels of depression, anxiety, stress, and hyperarousal, and the academic achievements of medical students were observed (Table 6). The grade point averages showed the most pronounced correlation with stress levels (*r* = −0.632, *p* < 0.01).

**Table 6:** Correlation analysis between educational performance among medical students of Western Ukraine with the level of depression, anxiety, stress traumatization subcomponents after 18 months of war.

Variable	*n*	*M*	SD	1	2	3	4	5
1.Grade Point Average	1,304	159.32	10.53	–				
2.Depression	776	6.74	4.90	–0.378**	–			
3.Anxiety	776	5.78	4.74	–0.485**	0.681**	–		
4.Stress	776	9.35	4.63	–0.632**	0.519**	0.487**	–	
5.Hyperarousal	491	1.61	0.37	–0.549**	0.292*	0.302*	0.265*	–

* *p* < 0.05; ** *p* < 0.01.

The multiple regression analysis (Table 7), with the level of student academic performance as a dependent variable, has shown that the academic achievements of medical students during the war were significantly related to the level of stress, depression, anxiety, and hyperarousal (*p* < 0.05). Interestingly, we have found that the direction of the relationship varies, showing a negative association with stress and a positive with hyperarousal, anxiety, and even depression. In contrast, the subscales of IES-R intrusion and avoidance did not show a significant influence on student academic achievements (*p* > 0.05).

**Table 7:** The multiple regression analysis of academic performance among medical students with depression, anxiety, stress, and hyperarousal subscales.

Predictor	Non-standard coefficients		95% CI		*р*-value
	*B*	Standard error, *B*	LL	UL	
Constant	167.614	1.869	165.321	169.510	0.000
Hyperarousal	2.375	0.601	1.857	2.712	0.000
Avoidance	−0.116	0.203	−0.187	−0.048	0.421
Intrusion	−0.204	0.147	−0.257	−0.155	0.311
Stress	−2.106	0.812	−2.516	−1.234	0.002
Depression	2.011	0.636	1.632	2.527	0.003
Anxiety	1.852	0.741	1.301	2.265	0.026

* *p* < 0.05.

## 4 Discussion

Wars always represent a threat to freedom, life, and health (19–22). Modern wars are especially aggressive because they use high-tech weapons to bombard territories regardless of distance. The Russian–Ukrainian war, ongoing since 24 February 2022, has caused human casualties and deaths and significantly affected the mental and physical health of Ukrainian citizens regardless of the region they live in.

The ongoing war enormously affects the education system, causing changes not only in the teaching processes themselves but also disrupting the main social, humanistic, and safe aspects of the learning environment (19, 20). In wartime, the education system must adapt to new realities and respond to relevant challenges while modifying the old and creating new models of academic interaction between students and teachers (3, 23). Depending on the distance between a higher education institution and combat clashes on the frontline, the war’s impact on the educational process may differ, which results in changes in the educational environment (19, 24), requiring different approaches. Nevertheless, maintaining physical and psychological health is key in all regions.

According to our research findings, medical student academic achievements during wartime were significantly lower than in the pre-war period (*p* = 0.000). The practical classes at the University in 2023 were conducted in a traditional face-to-face format. Even though medical students were studying in the rear region, we found that the war had a negative impact on their mental health, which, as we hypothesized was the main reason for the decline in student academic performance. The established levels of depression, anxiety, stress, and post-traumatic stress disorder affected the majority of students, and their symptoms ranged from moderate to severe.

Researchers emphasize that depression, anxiety, stress, and burnout are widespread among medical students (8–11, 23, 25). It has been hypothesized that this happens due to the very specifics of medical education with high responsibility and pressure for academic performance and heavy workload (compared to students of other specialties) during all years of study (25–27). However, the relationship of the war factor with all its consequences as a special stimulus that can cause psycho-emotional changes in medical students with mental health disorders has received relatively little attention.

We found that the prevalence of depression symptoms among university medical students after 18 months of war was 58.8%, significantly higher than the results of global meta-analyses (27.2–28%) and Asian countries (29.1–30.1%), respectively (4, 28–30). Regional distribution showed a high frequency of depression in the Middle East, with a prevalence of 31.8%, followed by North America – 30.3%, South America – 26.8%, and Europe – 20% (9, 26). Depression in medical students is accompanied by dysphoria, feelings of hopelessness, self-deprecation, and lack of interest (10, 31). In addition to depression, high levels of anxiety and stress are common among medical students (9). Our research defined symptoms of anxiety in 59.6% of university students, which is significantly higher than the 33.8% prevalence in a global meta-analysis and even higher compared to the regions with the highest prevalence of anxiety, such as 42.4% in the Middle East and 35.2% in Asia (11, 27).

The stress levels among university medical students showed the highest prevalence – 62.5%. Among the manifestations of depression, anxiety, and stress, symptoms of severe or extremely severe levels have been found quite common, with 25.6% of students showing signs of depression, 31.9% signs of anxiety, and 29.7% signs of stress, respectively. In contrast to prevalence data, quantitative characteristics were most expressed in stress and depression, and less in anxiety symptoms.

In non-war countries, lifestyle changes, financial stress, changes in family relationships, and academic concerns are among the factors that contribute to the development of depression (32–34). In contrast to these, the main factors for Ukrainian medical students in wartime are considered to be a constant threat to their life and health due to regular bombardment by missiles and unmanned aerial vehicles, even in the rear regions, as well as concerns about their relatives and friends, and shift in the educational environment from classrooms to civil defense shelters (3, 22).

Recent research of both prevalence and quantitative characteristics confirmed previous researcher findings regarding gender differences in the manifestations of depression, anxiety, and stress symptoms among medical students (8, 35). Moreover, our studies showed that the prevalence and severity of anxiety and stress differed significantly between male and female students. The researchers hypothesized that biological factors underlie the higher levels of depression, anxiety, and stress in female students, which may include hormonal profiles, neural circuitry, and neurotransmitter activity (9, 11, 23). These factors may also contribute to psychological characteristics predisposing to anxiety, neuroticism, increased interpersonal sensitivity, and attentional threat bias (29).

According to the IES-R scale, which is used to measure the stress reaction to traumatization situations, we determined high rates of PTSD symptoms among medical students, with a prevalence of 65.9%. Moreover, the average rates were high for all three subscales of post-traumatic stress disorder: intrusion, avoidance, and hyperarousal. The level of PTSD was more prevalent among female students compared to male students. High IES-R points indicated significant PTSD symptoms as the medical students experienced certain concerns during wartime in Ukraine, and some of them were indirectly related to the hostilities through relatives and friends (36).

Our data show that the levels of anxiety, stress, and depression are moderately inversely correlated with academic achievement. Among the IES-R components, hyperarousal, due to a reverse correlation, also negatively impacted student performance during their studies. The results of regression analysis indicated that the academic performance of medical students in the rear regions of Western Ukraine during wartime was directly influenced by the levels of hyperarousal, anxiety, and depression levels, as well as stress levels, which had an inverse effect. Some studies have shown that medical education itself may cause a certain level of anxiety and depression in students (10, 37). Researchers indicated that depression has not been defined as a negative predictor of academic performance. Based on their data, students achieve optimal performance levels when they experience minimal stress. However, another study (38) reported controversial results about the impact of anxiety and depression on academic performance, which require further studies. A large American study involving 5,551 undergraduate and graduate students found that individuals with high test anxiety, especially female students, had higher GPAs compared to those with low test anxiety (39). This finding aligns with our results, which also show a positive relationship between anxiety and academic performance. We hypothesize that the positive associations with depression, anxiety, and hyperarousal might represent a phenomenon where mild or moderate levels of these symptoms drive students to perform under pressure. Conversely, the negative association with stress more likely suggests a point where stress impairs performance. So, our findings suggest that students’ mental health and the level of PTSD development during the war in Ukraine significantly impact their educational performance, as well as the efficiency and functionality of the educational process.

Various types of student mental health promotion and prevention programs are becoming more common as universities begin to recognize the importance of mental health (10, 40–42). A survey of medical schools in the United States found that more than half of the respondents offered a curriculum dedicated to student well-being, with both compulsory and elective classes. These may include peer support, stress management and coping strategies, counseling, different physical activities, financial preparedness, scheduling improvement, recreational activities, and social events (10, 41, 42). The development of comprehensive wellness programs has to be focused on the student’s life experiences and real needs to ensure that the programs are effective and acceptable. For instance, Ternopil National Medical University has established a mental health center, conducts various trainings on mental health support, and has included an elective educational component called Mental Health Gap Action Programme in the curriculum.

## 5 Study limitations and future directions

Several limitations of the current study need to be acknowledged. First, the study was conducted at a single medical university in a specific region of Ukraine. Therefore, large-scale research that targets and compares different rear and frontline regions and medical universities is needed. Second, the use of cross-sectional design limits our ability to establish causality between the mental health outcomes and the student’s academic achievements, as we discussed associations, but not definitive cause-and-effect relationships. Future research should involve longitudinal follow-up with a large and more diverse participant group to assess the long-term impact of wartime stress on academic performance and psychological well-being. Third, our study focuses on the impact of war burden on medical students’ psychological well-being. However, other significant factors, such as the COVID-19 pandemic or pre-existing mental health conditions, may have also contributed to the outcomes. Unraveling their specific effects was beyond the scope of our current study. In the future, the relationships among these variables could be further explored and validated in longitudinal multi-center studies, along with the factors that positively affect mental health in medical students.

## 6 Conclusion

In summary, the results of the current study highlight the negative impact of the war in Ukraine on the mental health and academic performance of medical students. High levels of anxiety, stress, and depression contribute to the development of post-traumatic stress disorder, which emerged as a key predictor influencing the level of academic performance of medical students. The effectiveness and functionality of the educational process during the war plays an important role in ensuring the availability of skilled medical personnel and providing high-quality medical care to the population. Universities need to develop and implement more activities and programs aimed at supporting students’ mental health. These could include regular mental health screenings, offering accessible counseling services, and creating support networks that could help identify and address psychological issues. Moreover, universities could consider adapting curricula to reduce academic pressure in conflict areas and provide flexibility for students facing these stressors. By implementing effective measures to improve the mental health of medical students, universities can reduce the prevalence of PTSD and enhance both the quality of education and academic performance.

## Data Availability

The original contributions presented in the study are included in the article/supplementary material, further inquiries can be directed to the corresponding author.
